# Time to Cure Hairy Cell Leukemia

**DOI:** 10.4274/tjh.2017.0296

**Published:** 2017-12-01

**Authors:** Ilana Levy, Tamar Tadmor

**Affiliations:** 1 Bnai Zion Medical Center, Internal Medicine B Department, Haifa, Israel; 2 Bnai Zion Medical Center, Hematology Unit, Haifa, Israel; 3 Bruce Rappaport Faculty of Medicine, Technion, Haifa, Israel

**Keywords:** Hairy cell leukemia, Purine analogs, Cladribine, Pentostatin

## To the Editor,

Major advances in the treatment of hairy cell leukemia (HCL) occurred during the 1980s when the purine analogs (PAs) cladribine [1] and pentostatin [2] were introduced. These agents dramatically altered the clinical course and outcome of this disease, achieving a 10-year overall survival (OS) rate of approximately 90%. However, in the last 30 years, first-line therapy for patients with HCL and even second-line therapy have not been substantially changed.

In the current issue of this journal, Öngören et al. [3] report a retrospective analysis of 67 patients treated for classic HCL and compare 3 different first-line treatment modalities. Among them, 31 patients received cladribine therapy, 19 received interferon-alpha (IFN-α), 16 underwent splenectomy, and 1 was treated with rituximab monotherapy. Patients treated with a PA as first-line therapy achieved the highest overall response rate (ORR) and significantly longer progression-free survival (PFS) with the lowest relapse rate, but had a similar OS rate when compared to other treatment modalities. However, in terms of therapy-related complications, there was a high rate of infections, which were mostly bacterial, with the highest rate reported in the cladribine-treated group. These results are in line with previous reports indicating that IFN-α and splenectomy are much less frequently used now.

Pentostatin and cladribine are both equally recommended as first-line therapy, achieving equivalent efficacies. Both are associated with low rates of relapse or refractory disease (R/R) [4,5,6,7,8], indicating that HCL is potentially curable [5,7,8].

Nevertheless, HCL patients do relapse after PA therapy, and the rate or timing of relapse is associated with both complete remission (CR) and minimal residual disease (MRD) status [6]. Indeed, patients with HCL and MRD positivity have shorter treatment-free intervals than those in CR after PA therapy [8]. Moreover, for each consequent relapse, the response rate to retreatment with PA decreases [5,7].

Taking all the above into consideration, in the future we should encourage the development of novel combinations or the use of consolidation therapies after the first response with PA is achieved, particularly in those patients who are MRD-positive, in an attempt to achieve more durable responses.

Another disadvantage that should be carefully considered is the high rate of PA toxicity, which includes bone marrow suppression associated with neutropenia, lymphopenia, T-cell dysfunction, or stem cell toxicity with the development of hypoplasia and aplasia [5,6,9,10]. Similar complications were also described in the study by Öngören et al. [3].

Novel agents with a lower toxicity profile are currently being tested as alternative therapy or in combination with PA. Rituximab, a monoclonal antibody, has been studied as monotherapy or in combination with PA, both in the frontline setting or at relapse [11]. Results are encouraging, and rituximab seems to be well tolerated while side effects are quite rare.

Other agents are also being used now for R/R HCL patients. These drugs are not used as frontline therapy but have a favorable toxicity profile. These include recombinant immunotoxins targeting CD22 (BL22, moxetumomab pasudox) [5] or the BRAF inhibitor (vemurafenib) [4] and have shown positive results in R/R disease [5]. Finally, the Bruton tyrosine kinase inhibitor ibrutinib appears to shorten the survival of hairy cells and block cell proliferation and intracellular signaling in vitro [13], and it is effective in some preliminary reports of therapy in refractory disease [14]. However, in this respect, due to the rarity of the disease, data are still incomplete and mostly based on small case series, retrospective studies, or phase I clinical trials [12,13,14,15].

Based on the recently published HCL consensus by Grever et al., [6] PAs remain the only first-line therapy for HCL, while vemurafenib is recommended in some cases with uncontrolled infection prior to therapy with PAs due to its ability to improve low blood counts. However, this agent should be replaced by a PA as soon as the infectious status is controlled. In regard to relapsed disease, both vemurafenib monotherapy and rituximab in combination with PA may be recommended, although repeated PA therapy is preferred in patients who achieve long first remissions of more than 60 months [6]. Other novel therapies such as ibrutinib or immunotoxin conjugates are still mostly used in clinical trials and are not included in current guidelines.

In conclusion, as reported by Öngören et al. [3], PAs remain the most effective treatment for classic HCL in terms of PFS and ORR when compared to earlier therapies such as IFN-α or splenectomy, and this approach has not been changed over the past 30 years. Perhaps the time has arrived to challenge this approach and improve the outcome of HCL by using PAs in combination with some of the available biological agents, either as frontline or consolidation therapy for patients with classical HCL, in an attempt to cure this chronic neoplastic disorder.
